# Indoor Air Complaints: VOCs May Not Be Cause of Acute Effects

**Published:** 2005-11

**Authors:** Bob Weinhold

Over the past few decades, researchers have been trying to pin down the specific chemical culprits behind increasing complaints of poor air quality inside offices and other buildings. Among the many chemicals suspected so far have been volatile organic compounds (VOCs) and ozone, prominent pollutants in indoor environments. But VOCs alone, or in combination with ozone, may not be the prime source of acute health problems, says a team of New Jersey investigators **[*EHP* 113:1542–1548]**. Instead, they found that psychological stress was a more salient factor, but they acknowledge that a number of limitations in their study preclude applying this finding to all indoor air complaints.

The study investigated the short-term acute health effects of exposure to ozone, a mixture of 23 VOCs, and stress. The research was conducted in a controlled chamber into which either a relatively high level of the VOC mixture (26 milligrams per cubic meter), the VOCs plus moderate concentrations of ozone (40 parts per billion), or clean air with a low one-minute spike of VOCs (about 2.5 milligrams per cubic meter) was introduced. In the middle of each three-hour test session half of the volunteer subjects had to make a four-minute speech on a controversial subject as a stress test, while the other half performed simple arithmetic problems. The test sessions were held one week apart.

The researchers evaluated stress by measuring cortisol secretions in saliva. To assess health effects, they evaluated selected performance measures, as well as 33 observed and self-reported physical and behavioral indicators, such as headache, nausea, eye irritation, nervousness, and leg cramps.

They found the challenge of public speaking induced a significant increase in the subjects’ measures of stress. However, even with that increase in stress, no significant increase in health symptoms or reduction in neurobehavioral performance was linked to the exposures to VOCs either alone or combined with ozone, despite sharp increases in many secondary pollutants resulting when ozone was added to the VOC mixture.

The 130 female volunteers exposed to each air mixture constituted the largest group evaluated in a study of this kind, and the researchers determined the numbers were of sufficient power to produce significant findings. However, all the subjects were healthy, young (mean age 27.2 years), and well educated (mean education of 15.2 years), demographically limiting the applicability of the findings.

In addition, the team acknowledges that its testing, while extensive, didn’t represent many aspects of a typical office building. For instance, the test chamber did not include carpet, many office furniture materials, and other normal interior accoutrements that might interact with VOCs and ozone. The mix of VOCs, although extensive, likely didn’t represent the mix in many buildings. Further, the ventilation rate in the test chamber was substantially higher than in many buildings at which complaints have been lodged.

Further, the public speaking challenge, although successful at inducing stress, wasn’t representative of the multiple complex stressors experienced in a typical work day. And the testing period was very short, providing no information on the potential chronic effects that may be induced by longer-term exposures to chemicals and stress. Nonetheless, the findings are helpful in pinning down the relative contribution, or lack thereof, of certain mixtures and concentrations of VOCs and ozone to poor indoor air quality.

## Figures and Tables

**Figure:**
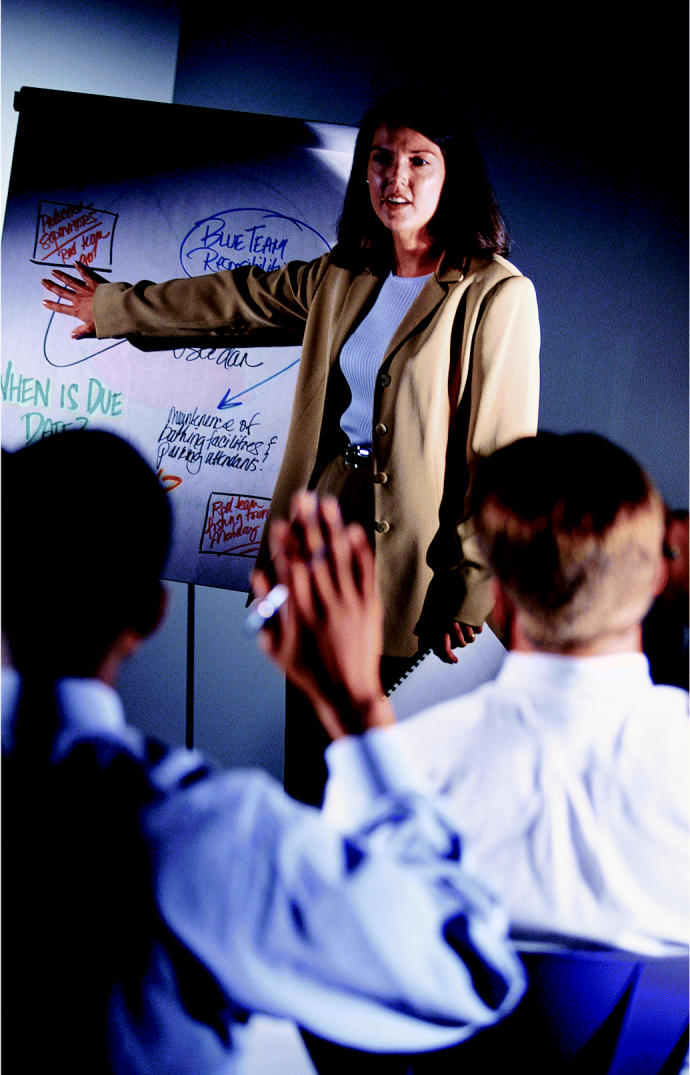
Maybe it’s nerves. A study of indoor air pollutants and stress (induced by public speaking) shows that stress, not VOCs, may be a larger culprit behind sick building complaints.

